# Medical Schools and Medical Students

**Published:** 1888-09-15

**Authors:** 


### MEDICAL SCHOOLS AND MEDICAL STUDENTS.

### A PARENT'S PROTEST.

I NEED hardly tell you how much interested I am in that capital little paper, THE HOSPITAL, which I read attentively and then send to a young medical man in Canada. I know what an active mind and incisive pen you have, and I am sure you will excuse me for making a suggestion which, if you could see your way to carrying it out through THE HOSPITAL, might be of much importance, not only to parents, but also to the medical profession. It appears to me that the several medical schools do everything in their power to collect a number of medical students. They probably think that out of the number they will get sufficient for the service of the hospital from those who can and will work alone, but they do not seem to care a farthing about the others. An idle youth may do almost nothing, provided he does not appear drunk in the hospital. There is nothing to ensure their attendance, not even an attendance book, and when in hospital they may do (or not do) pretty well as they like and leave when they please. This seems to me very unfair to parents who have to pay fees for boys who are under little or no control, and they have to fish out the times for their examinations as best they can. It is also unfair to the medical profession and to the public, for at the last moment youths cram for the examinations. They scrape through, and many of them become most inefficient medical practitioners and kill half their patients. This, of course, throws the medical profession into disrepute. You will possibly say, "What remedy do you propose?" I am only a layman, and therefore think that you and your medical advisers would be better hands at finding a remedy than I could possibly be. But I have little doubt that parents would gladly pay more than they now do if they felt that their sons were: (1.) Under some supervision. (2.) If they had to note their arrivals and departures in an attendance book. (3.) If they were not allowed outside the hospital walls, except during specified hours. (4.) If they had to assemble in a room for a certain number of specified hours, and a medical officer paid for hearing them their medical lessons and noting the results in a book to be submitted to a superior medical officer who should act on the principle *disce aut discede.* A small college attached to each medical school, with a charge for board, lodging, and supervision, would be of immense value, but a good deal might be accomplished without that. Now forgive me if I have made a stupid suggestion, and with my kind regards, I am, &c.

The Glebe, Lee, S.E. FRED. CLEEVE.

### WHAT THE DEANS SAY.

### From Frederick Taylor, M.R.C.S., Dean of Guy's Hospital Medical School.

I CANNOT express any opinion on the suggestions contained in Mr. Cleeve's letter without first alluding to the exaggerated and incorrect statements made in the first part of it. In particular, I am quite sure that he has no justification for assuming that the authorities of the medical schools are guided in their management of the students solely by a desire to get men for the service of the hospital, and that they care not "a farthing about the others"; nor for his statement that many youths "become most inefficient medical practitioners, and kill half their patients." But in spite of these inaccuracies, Mr. Cleeve may be able to make some good suggestions for the management of our students. Let us see: Firstly, he thinks that they should be under some supervision. With this I entirely agree. But then, they are already under supervision. Their attendance on lectures is rewarded; their attendance at practical work — dissecting, chemistry, histology, bandaging, etc., is directly under the observation of the teachers; the fulfilment of certain duties in the wards and out-patient departments is rendered obligatory by the examining boards, and is constantly checked by the officers of the hospital to whom the students are appointed. But Mr. Cleeve's suggestion goes further, the second and third being really only the details of a closer supervision than is commonly carried out. My own opinion is that the second arrangement—to have an attendance-book—would be futile; and the third—to keep the students within the walls, like schoolboys—would be impracticable. The attendance-book would soon become a farce; and whether the students would benefit or not by being kept within the precincts of the hospital would depend upon the work they were at the time engaged upon. As a matter of fact, we have no power to retain them. With regard to the fourth suggestion, that the students should "assemble in a room for a certain number of specified hours, and a medical officer should be paid for hearing them their medical lessons and noting the results in a book, to be submitted to a superior medical officer, who should act on the principle *disce aut discede*," I would remark that the system is more or less carried out already in the several subjects of education, though not quite in such a drastic manner as Mr. Cleeve seems to propose. The student is habitually taught by frequent questions on the part of his teacher. Whether this is done in one room or another, for one, two, or three hours, or how many students are together at one time must surely be left to the experience of the teacher and the requirements of each particular subject. And I can assure Mr. Cleeve that the expulsion of a student (for surely that is what he means by *disce aut discede*) is a proceeding which requires much more delicate handling than his proposed arrangement provides for. Mr. Cleeve does not seem to realize that our students are not schoolboys, and cannot be treated as such. Many of them are men of 25 or 30 years of age, or more. We have no direct means of punishment, other than suspension or expulsion. The first defeats its object; the second can scarcely be employed to punish idleness, unless it is persistent and incorrigible. If a student is indifferent to his future, and indifferent to the wishes of his parents or guardians, mere supervision will not drive him to work. If a student is dull and badly trained, supervision will only get him slowly on. But supervision should, of course, be exercised; and it is exercised in the school to which I am attached, and, I believe, in every other medical school in the kingdom, to a much larger extent than Mr. Cleeve seems to think. So far as this object can be assisted by a residential college, I am glad to say that we shall be able shortly to fall in with Mr. Cleeve's views, as arrangements are now on foot for the erection of a residential college in connexion with Guy's Hospital and its medical school.

### From A. Pearce Gould, F.R.C.S., Dean of Middlesex Hospital Medical School.

I can, of course, answer only for my own medical school, but, so far as that is concerned, Mr. Cleeve's strictures are in no way applicable. We do care a great deal about all our students, and we do not allow idle students to remain indefinitely with us. An exact record is kept of the attendance of each student at all classes, and their entire curriculum is planned out for them, and they are required to follow it in all details. So far as the parents are concerned, we state in our prospectus exactly when each examination ought to be passed, and twice a-year we send them a report of the class attendances of their sons. Thus each one of Mr. Cleeve's grave charges is incorrect, as applied to this hospital. I do not think his suggestions are practicable. I believe that all that can be done is to see that each student attends his classes and pursues his practical studies regularly and diligently, and by advice and counsel aid those who are falling behind in their work. Our residential college is a great success, and supplies a much-felt want.

### From John Carson, M.D., Dean of the Medical Faculty King's College.

Our students are under supervision, and reports are sent to their parents and guardians at the end of every term. The other suggestions are quite impracticable, and would not have been made if Mr. Cleeve were fully acquainted with the curriculum and conditions of medical work.

### From A. H. Young, M.D., Dean of the Medical Faculty, The Owens College, Manchester.

You are good enough to ask for my opinion on the suggestions, etc., contained in the letter of Mr. Fred. Cleeve, referring to medical schools and medical students. Mr. Cleeve's comments upon the objects which medical schools have in view in seeking to extend their field of work and attract larger numbers of students are, in my opinion, both unjust and ungenerous. It is somewhat unfortunate that he does not clearly distinguish between a medical school and the hospital with which it is connected. I can, of course, only speak with any authority of what happens in a provincial school of medicine; but of this I am sure, that there is no such carelessness and apathy as to the fate of students, who either do not or cannot work, as Mr. Cleeve supposes. In the medical department of Owens College, if a student neglects his lectures or practical work, his parents are communicated with, whenever possible, and a report is forwarded to them. The regular attendance of students is insisted on, a daily record is kept, and examinations are frequently held. In the associated hospital the attendance of all students on lectures, ward classes, demonstrations, etc., is recorded, and must be regular. Dressers and clerks were formerly required to enter their names in an attendance book, but this has been abandoned as practically useless. We have in Manchester the advantage of two excellent "halls of residence" connected with the Owens College. Still a large number of the students live at home or in private apartments. It is unusual in any medical school that the students, as a body, reside in the hospital, and it is clearly impossible to refuse to allow them "outside the hospital walls except during specified hours." I have already shown that in Manchester a considerable amount of supervision is exercised. So also, I believe, is the case in all important medical schools. But it is quite true that if any individual student devotes himself to the task of evading work, transgressing regulations, and cheating the authorities, he may succeed just as well as he not unfrequently does at home, even when under the direct and personal supervision of his parents. The fault is not always in the management of the medical school, and it should not be forgotten that students vary in age, in ability, and in character. To me it seems unfair that those who work—and in my experience they form a very large majority—should be made subject to irksome, irri tating, and stringen regulations because some two or three habitually neglect to make any use of opportunities freely offered to them. Mr. Cleeve has apparently been singularly unfortunate in meeting with students of the latter class, and one wonders what medical schools he is most familiar with. If it be true, as he states, that such students "cram" up for examinations at the last moment, scrape through, become indifferent practitioners, and kill half their patients, it seems to me that he has a very strong case in favour of the reform of the present examination system. I cannot, however, quite accept Mr. Cleeve's statements on these points. It may be that Mr. Cleeve, in his letter, only intended to refer to young students who commence their medical studies with a view to obtaining a qualification at the earliest possible age, and with the devotion of the least possible time to the necessary curriculum, since he only refers to students as "boys and youths." I would point out that under these circumstances the dangers which he fears are real, but this only affords me the opportunity of suggesting the extreme desirability of parents allowing their sons to devote at least a year to those preliminary scientific studies which are of such extreme importance to the scientific medical practitioner. By this means habits of study are acquired, whilst the character of the "boy" becomes more matured.

### From Mr. H. W. Mackintosh, Registrar of the School of Physic, Trinity College, Dublin.

While there is much in Mr. Cleeve's comments on medical schools and students with which I agree, I am bound to take exception to what I may perhaps call the manner in which he presents his subject. Anyone unversed in such matters, reading Mr. Cleeve's remarks, would, I think, certainly infer that all medical schools stood on the same level, and were organised on the same lines. As you very justly observe in your letter to me, his criticisms do not in any sense apply to schools connected with colleges which are manned by a staff of teachers, some at least of whom give up their whole time (practically) to the working of their own departments, and exercise strict supervision over the students who work under them. I have no doubt that Mr. Cleeve would at once admit the distinction, which, I venture to think, is an important one, between such schools which I hold to be the true medical schools, and the mere gatherings of embryo doctors round a hospital where little or no systematic instruction is given, which are too often dignified by the name of medical schools. It is upon these, if I understand him aright, that Mr. Cleeve empties the vials of his indignation; and I entirely concur with him, objecting, meanwhile, to his not having made the difference sufficiently clear. For my own part, I would go much further than Mr. Cleeve proposes, and would adopt a much more drastic line of treatment with the majority of these hospital schools, the existence of which, I believe, to be a very serious defect in our medical education system. I have little doubt that all Mr. Cleeve says is true of many of them, and that a great deal more might be said to their dispraise; and so far from trying to bolster them up as Mr. Cleeve seems inclined to do, I would root them up bodily, either by having the various licensing corporations refuse their certificates, or an intelligent and properly-informed public opinion refuse to send students to them, thus concentrating medical teaching in a smaller number of properly-equipped and organised institutions (call them of what name you please), in which a full curriculum would be superintended by able men giving up a considerable proportion of their time and care to the work. For various reasons I am inclined to think that the general refusal of such certificates by licensing bodies is a state which pertains to Utopia, and which we are therefore little likely to arrive at; but I would expect a considerable measure of success in endeavouring to enlighten parents and guardians as to the vast differences which obtain between different medical schools, using the term in its widest sense. "I have little doubt," says Mr. Cleeve, "that parents would gladly pay more than they now do if they felt that their sons were under some sort of supervision." I think facts go to show that Mr. Cleeve is quite right, and that parents who can afford it, and who take the trouble to investigate the subject a little, always do send their sons to schools where they are properly supervised, even though they have to "pay more." It seems to me that in a very large proportion of instances parents are themselves culpable, for they make no attempt to get any information beforehand for themselves, but either send their sons to some place where a chum is studying, simply because the boy would naturally vote for being with his chum, or else take the opinion of the family doctor or the schoolmaster, who very often know nothing about the subject. The evil is widespread and deep-rooted, drawing sustenance from very many sources, and sometimes one is inclined to think that the prospects of even an amelioration, not to say a cure, are too remote to be worth trying for; it involves sound education all round. With regard to Mr. Cleeve's four suggestions, I would say that I agree so fully with (1), that I would suppress all "schools in which there was no supervision;" (2) I think is impracticable, unless an official could be obtained to take charge of an attendance-book, who never left his post, who watched each entry, and who knew each student personally, and even then I do not know that this kind of supervision is at all desirable; (3) I think would only secure an appearance of diligence without any necessary reality, and would be an irritating restriction on many of the hard-working men; (4) I think is mistaken in principle, and would prove faulty in practice. It seems to me that it tends to maintain the schoolboy spirit, instead of developing the sense of personal responsibility which, in my judgment, is an indispensable part of education, and to substitute the mere learning of lessons for the habit of observation and reasoning, which a man must acquire or he will never be a good doctor, while in practice it commits far too important a decision to the hands of the "superior medical officer," who would need to be very superior indeed, both in knowledge and in principles, to carry out strictly and fairly the rule of *disce aut discede*, an excellent rule, and one which I would gladly see in operation in every school and college, but requiring great care and patience in its application, which should be in the hands, not of an individual, but of a court or board. It seems to me the true remedy is to be had in raising the standard of medical education; but to discuss this side of the question would take up far too much of your time. I have already encroached on it to an unpardonable extent. In conclusion, permit me (1) to say that, widely as I differ from Mr. Cleeve in many of his remarks, I think he deserves the thanks of all who are interested in medical education for directing attention to the subject; (2) to thank you for your courtesy in waiting my convenience to give you my view of Mr. Cleeve's suggestions, which you are very welcome to publish if you think fit; (3) to express my regret that I have not written something better worth the trouble of reading.

**From John William Moore, M.D., Fellow and Registrar of King and Queen's College of Physicians, Ireland.**

I have to acknowledge the receipt of your letter of June 1st, enclosing a letter from Mr. Fred Cleeve, C.B., J.P., in which he embodies certain suggestions as to an increased supervision over medical students. In attempting to express my opinion of Mr. Cleeve's suggestions, it is necessary to remind you at the outset that the relations which in London exist between hospitals and medical schools do not hold good in Dublin. Students are free to select a hospital and school which are quite independent of each other. Indeed, in no one instance have we in Dublin a complete establishment of hospital and school, under the same management and officered by the same staff. This fact disposes of at least one of Mr. Cleeve's suggestions, viz., that medical students should not be "allowed outside the hospital walls, except during specified hours." It also disposes of his ungenerous—I would add reckless—statement, that "the several medical schools do everything in their power to collect a number of medical students. They probably think that out of the number they will get sufficient for the service of the hospital from those who can and will work alone, but they do not seem to care a farthing about the others." I cannot believe that this charge is true of the medical schools of London, any more than it is of those of Dublin. Mr. Cleeve, in his letter, goes on to speak of medical students as "boys who are no under little or no control." Surely this is not a fair description of young men who must be at least of legal age at the time when they are permitted to receive their qualifications to practice. He adds: "At the last moment youths cram for the examinations. They scrape through, and many of them become most inefficient medical practioners and kill half their patients." This strong language refutes itself. But I assert that, under the regulations for the qualifying examinations in medicine, surgery, and midwifery held by the King and Queen's College of Physicians and the Royal College of Surgeons in Ireland, it is simply impossible to pass on mere "cram." For your information, and for that of Mr. Cleeve, I beg to forward copies of the regulations for the conjoint examinations of the Irish Royal Colleges. And now as to Mr. Cleeve's other suggestions. He asks for "supervision" over medical students. This I maintain we at present have to a reasonable extent, always recollecting that we have to deal with young men who have already deliberately chosen one of the most solemn and responsible callings in life. Surely, under these circumstances, it should be sufficient to require a daily punctual attendance at the hospital and medical school in the character of gentlemen. He suggests again that students should be obliged "to note their arrivals and departures in an attendance-book." Well, this plan has been tried, and over and over again it has proved wanting. It leads to great abuses, and can never effect the object in view. Men who are alive to the reality of their student work will attend daily and in time. Men who are not cannot be made so to attend by such means as Mr. Cleeve suggests. The idea of a "medical officer paid for hearing students of from 17 to 21 years of age their medical lesson" is almost grotesque, and may be dismissed from consideration. Lastly, I do approve Mr. Cleeve's suggestion as to residence houses attached to medical schools in certain cases and for certain students. Kindly regard this letter as non-official, and simply embodying my personal opinion.

